# Conductive Metal-Organic Frameworks for Amperometric Sensing of Paracetamol

**DOI:** 10.3389/fchem.2020.594093

**Published:** 2020-12-08

**Authors:** Jing Wang, Sen Liu, Jiahuan Luo, Shaogang Hou, Haixiang Song, Yongsheng Niu, Chuanxiang Zhang

**Affiliations:** ^1^Henan Joint International Research Laboratory of Nanocomposite Sensing Materials, School of Chemical and Environmental Engineering, Anyang Institute of Technology, Anyang, China; ^2^Henan Key Laboratory of Coal Green Conversion, College of Chemistry and Chemical Engineering, Henan Polytechnic University, Jiaozuo, China

**Keywords:** sensor, conductive MOF, NiCu-CAT, amperometric, paracetamol detection

## Abstract

An electrochemical sensor for paracetamol is executed by using conductive MOF (NiCu-CAT), which is synthesized by 2, 3, 6, 7, 10, 11-hexahydroxytriphenylene (HHTP) ligand. The utility of this 2D NiCu-CAT is measured by the detection of paracetamol, p-stacking within the MOF layers is essential to achieve high electrical conductivity, redox activity, and catalytic activity. In particular, NiCu-CAT demonstrated detection Limit of determination near 5μM for paracetamol through a wide concentration range (5–190 μM). The NiCu-CAT/GCE exhibits excellent reproducibility, stability, and interference for paracetamol.

## Introduction

Paracetamol (PA) is a high effective antipyretic and analgesic drug, which is generally used to relieve moderate pain, such as headache caused by influenza or joint pain, migraine, etc. It regulates the synthesis and release of central prostaglandins by controlling the body temperature in the hypothalamus, improves the pain threshold and plays the role of antipyretic and analgesic (Wan et al., 2009; Ghadimi et al., 2013). The normal dose of paracetamol is harmless to the human body, but excessive or long-term use will lead to liver poisoning, leukemia, or even central nervous system poisoning (Fan et al., 2011). Therefore, it is necessary to develop a sensitive, simple, and rapid detection technology for paracetamol. The existing detection methods include spectrophotometry, titration analysis, chemiluminescence, capillary electrophoresis, fluorescence spectrum, high-performance liquid chromatography (Easwaramoorthy et al., 2001; Bosch et al., 2006). However, it is not suitable for the rapid detection of PA in daily life due to the complex pretreatment, large equipment, time-consuming and expensive of these existing detection methods. On the contrary, electrochemical methods have the advantages of being simple and convenient, highly sensitive, quickly responsive, low in cost and highly selective (Yan et al., 2019; Yuan et al., 2019, 2020; Ma et al., 2020; Wang et al., 2020). In addition, PA is an electroactive substance that is prone to electrochemical oxidation, so the detection of PA by electrochemical sensors has aroused great interest. However, the redox reaction of PA on the bare electrode is slow. As a result, researchers have developed high catalytic activity nanomaterials for the design of highly sensitive paracetamol electrochemical sensors (Ejaz and Jeon, 2017; Raymundo-Pereira et al., 2017; Zhao et al., 2019).

In recent decades, nanomaterials have attracted worldwide attention and have been widely used to modify electrochemical sensing electrodes. As one of the most popular materials at present, the conductive nano-Metal Organic Framework (MOFs) (Campbell and Dincă, 2017; Biswas et al., 2020; Ko et al., 2020; Suwannakot et al., 2020) have many advantages, such as simple synthesis process, environmental friendliness, adjustable structure and so on, especially its excellent conductivity, which has attracted extensive attention (Ko et al., 2017; Fang et al., 2018; Xie et al., 2020). Metal-catecholates (M-CATs) are a kind of conductive MOF composed of HHTP ligands and central metal ions (Miner et al., 2018; Zhang et al., 2018, 2019; Guo et al., 2019). The good conductivity of M-CATs is mainly due to its special structure, in which oxygen atoms in an HHTP ligand can also combine with axial water ligands to form hydrogen bonds. M-CATs show two accumulation modes: one kind, a two-dimensional layered framework with a hexagonal hole and honeycomb structure, is formed by oxygen and p-p interaction, where metal nodes and organic ligands serving as charge carriers enable full charge delocalization in the two-dimensional (2D) plane, so as to produce good electrical conductivity, and the other is along the c axis through hydrogen bonding accumulation, which is easy to form one-dimensional (1D) structures between layers. Because of their special porous structure and good electrical conductivity, M-CATs have great prospects for related applications such as catalysis, supercapacitors and electrical analysis.

In this paper, the 2D conductive nano-MOFs are first systematically studied for the detection of PA in multi-component aqueous solutions. The sensor has the characteristics of fast electronic transfer, good catalytic performance, and good detection limit of determination for paracetamol. Hence this work opens up a new method for electrochemical detection of paracetamol, which is beneficial to the study of the redox metabolism of paracetamol in aqueous solution and expands the application of MOF nanomaterials in electroanalytical chemistry.

## Methods and Materials

### Materials and Reagents

The chemical 2, 3, 6, 7, 10, 11-heahydroxytriphenylene (HHTP) was purchased from Innochem Reagents (Shanghai, China); Nickel (II) acetate tetrahydrate (Ni(OAc)_2_·4H_2_O), Copper (II) acetate tetrahydrate (Cu(OAc)_2_·4H_2_O), standard paracetamol, dopamine (DA), and ascorbic acid (AA) were purchased from Aladdin (Shanghai, China). All other chemicals were analytical reagent grade. Deionized water was prepared from a Milli-Q water purification system. Different pH phosphate buffers (0.1 mol L^−1^) were prepared by mixing KH_2_PO_4_ (0.1 mol L^−1^) and Na_2_HPO_4_ (0.1 mol L^−1^) solutions.

### Instrumentation

All chemicals were obtained from commercial sources and used without further purification. Powder X-ray diffraction (PXRD) data were collected on a Rigaku D/max-2,550 diffractometer with CuKα radiation (λ = 1.5418 Å). The infrared (IR) spectra were recorded within the 4,000–500 cm^−1^ region on a Nicolet Impact 410 FTIR spectrometer with KBr pellets. TEM, HAADF-STEM, HRTEM, and EDX were carried out on a FEI Talos F200S TEM (200 kV). The structure for NiCu-CAT was simulated by Materials Studio 8.0 and using the Crystallographic Information File (CIF) of Ni-CAT (Ko et al., 2020) as mode.

### Synthesis of the NiCu-CAT Nanocomposite

Typically, 30 mg of Ni(OAc)_2_·4H_2_O, 20 mg of Cu(OAc)_2_·4H_2_O, and 42 mg of HHTP ligands were dissolved in 9 mL of a solvent mixture of deionized water. The vial was capped and sonicated for 30 min until the solid was dissolved, and after that 0.5 mL of NMP was added drop-wise into this solution, and then this solution continue was sonicated for 10 min, the reaction mixture was transferred into an isothermal oven at 85°C for 12 h. After the crystals were washed with deionized water and acetone, the NiCu-CAT was obtained.

### Preparation of the Modified Electrodes

First of all, glassy carbon electrode (GCE) was mechanically polished on a velvet cloth with 0.05 μM alumina slurry. Secondly, then electrochemical polishing was carried out in a potential window of 0–1 V at a scanning speed of 100 mV/s in 0.1 M H_2_SO_4_. Thirdly, GCE was ultrasonicated in deionized water and ethanol for 5 s respectively. One milligram of NiCu-CAT was dispersed in 1 mL of distilled water and Nafion (5 wt%) and ultrasonicated by cell disrupter for 30 min to ensure a uniform dispersion. Then, 6 μL of this dispersion was dropped on cleaned GCE and dried at room temperature to obtain a suitable coating (NiCu-CAT/GCE).

### Electrochemical Measurements

Cyclic voltammetry (CV), electrochemical impedance spectroscopy (EIS) and differential pulse voltammetry (DPV) were carried out with a CHI760E electrochemical workstation (Shanghai Chenhua Instrument Co. Ltd., China) using a three-electrode system, with a bare or modified glassy carbon electrode (GCE, 3.0 mm in diameter) as the working electrode. A platinum (Pt) wire and Ag/AgCl were the counter and reference electrodes, respectively. All electrochemical experiments were conducted at room temperature (25°C).

## Results

### Characterization

**Figure 1:**
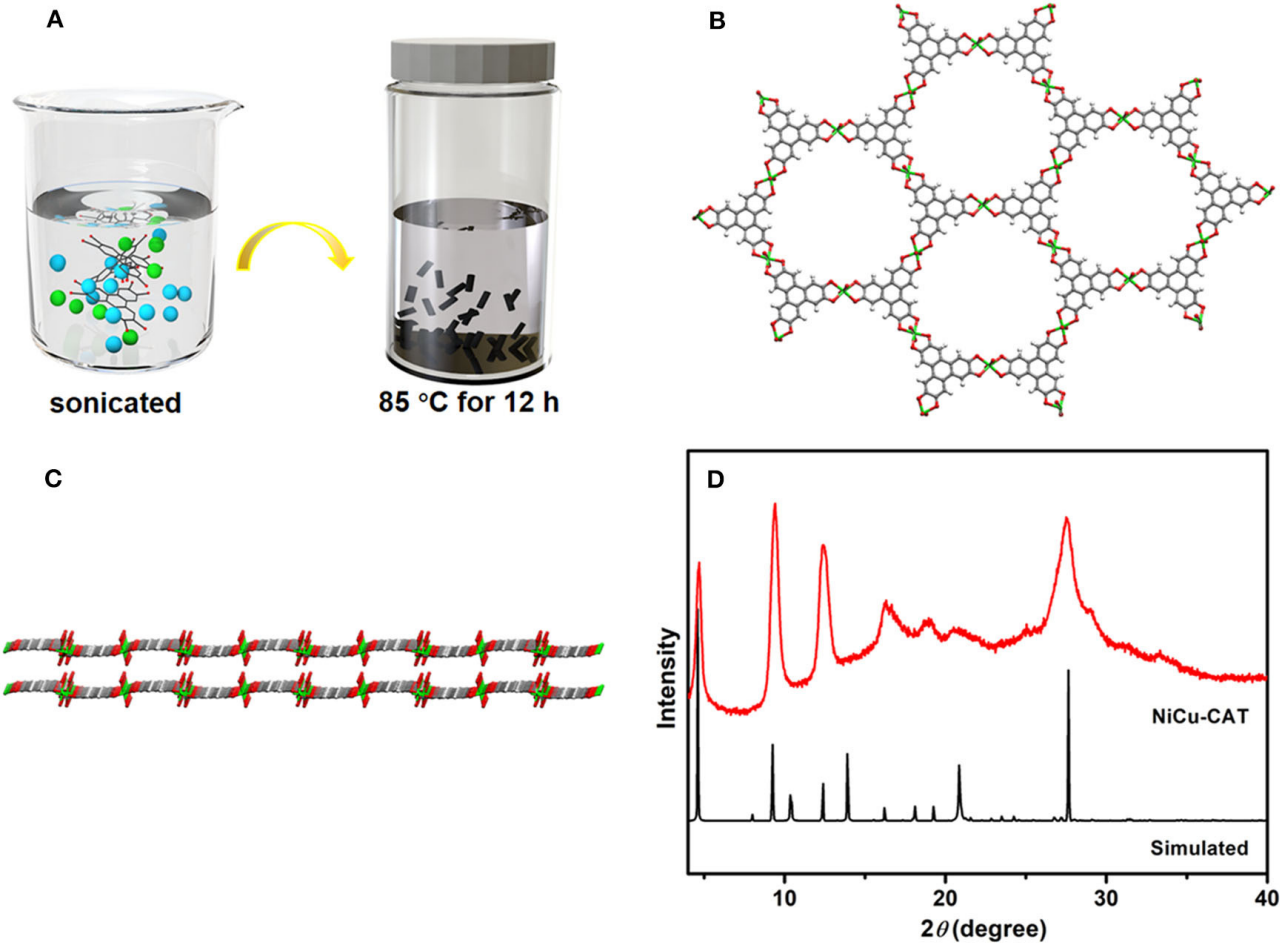
**(A)** Schematic illustration of the preparation of NiCu-CAT. **(B)** Connecting mode of HHTP molecules and Ni^2+^ and Cu^2+^ ions. **(C)** View of the two extended corrugated layers along the [110] direction. **(D)** XRD pattern of NiCu-CAT nanocrystal and simulated.

Typically, NiCu-CAT constructed from a 2D hexagonal lattice in the *ab*-plane, which configurations along the [001] direction, are synthesized by solvothermal method (Figure 1A). As shown in Figures 1B,C, the divalent metal ions (M^2+^) are matched to adjacent deprotonated HHTP ligands to form an extended 2D P-conjugation honeycomb framework. Each ligand can be oxidized to achieve charge balance with the metal ion centers, which is very important to improve the charge density of the M_3_(HHTP)_2_ (H_2_O)_12_ (Supplementary Figures 1a,b).

The X-ray diffraction (XRD) patterns of NiCu-CAT that matched reported characterization are shown in Figure 1D. The XRD pattern of NiCu-CAT clearly reveals three sharp intense peaks of (100), (200) and (210) planes at 2θ = 4.7°, 9.5°, and 12.4°, respectively. The peaks indicate the long-range order of the nanocrystal in the *ab*-plane (Miner et al., 2018; Guo et al., 2019; Ko et al., 2020). Fourier transform infrared spectroscopy (FT-IR) is conducted, as showed in Supplementary Figure 2. Typically, the bonds at 1,118, 1,430, and 3,099 cm^−1^ are attributed to the -C = C- stretching, -C-O- stretching vibration, and -O-H, respectively. The bands at 672 and 804 cm^−1^ represent the out-of-plane C-H bending modes, fully manifesting the existence of organic HHTP.

**Figure 2:**
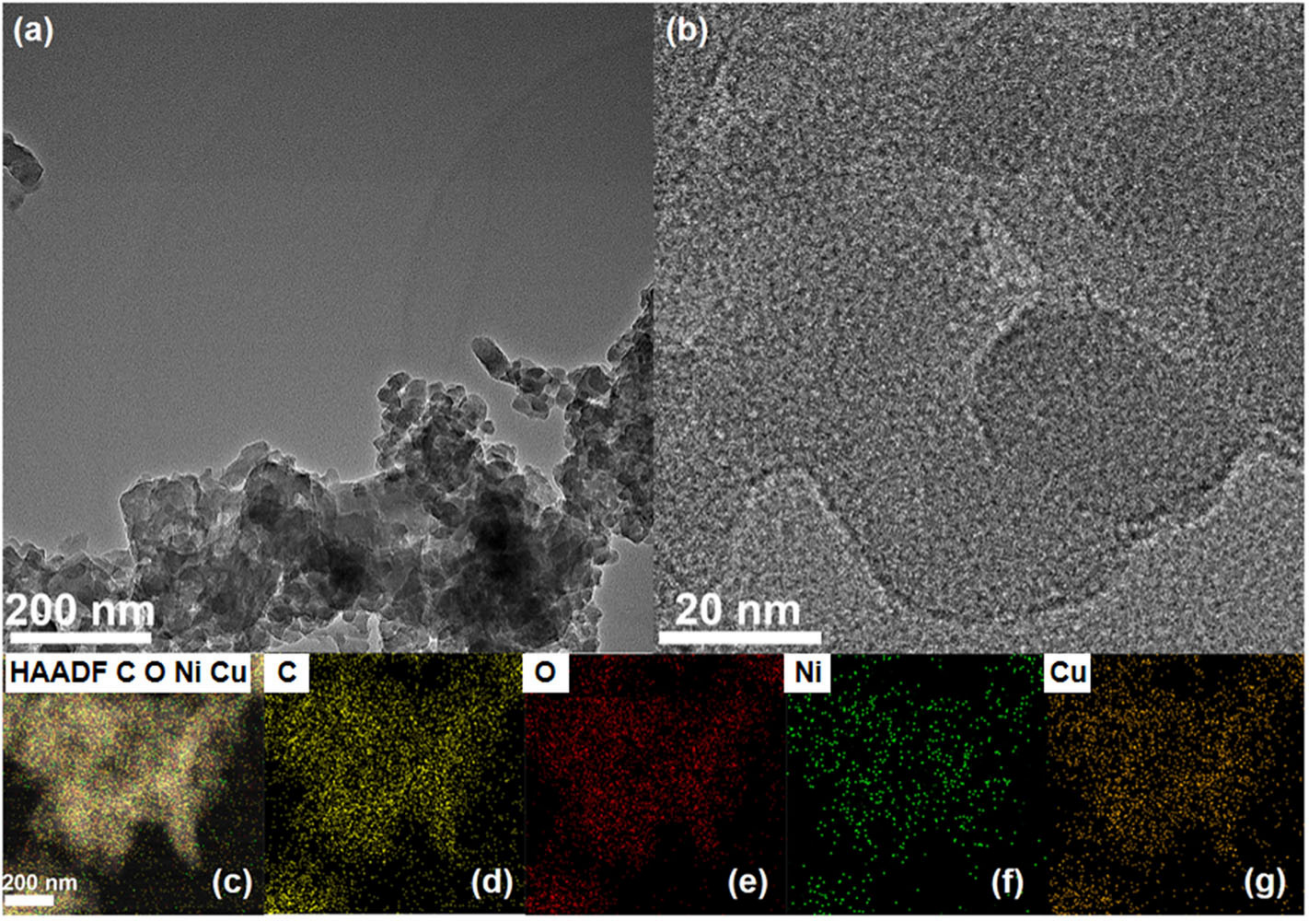
**(a,b)** TEM images of the NiCu-CAT nanocrystal. **(c–g)** The distribution images of various elements of NiCu-CAT nanocrystal.

Transmission electron microscopy (TEM), high-angle annular dark-field scanning TEM (HAADF-STEM), and high-resolution transmission electron microscopy (HRTEM) were executed to characterize the morphology of NiCu-CAT. As shown in Figures 2a,b, NiCu-CAT is made up of nanocrystals with the diameter of 20–50 nm. The corresponding energy dispersive X-ray spectroscopy (EDX) mapping images (Figures 2c–g and Supplementary Figure 4) evidence the even distribution of elemental Ni, Cu, C, and O throughout the nanocrystal.

### Effect of the pH Values

**Figure 3:**
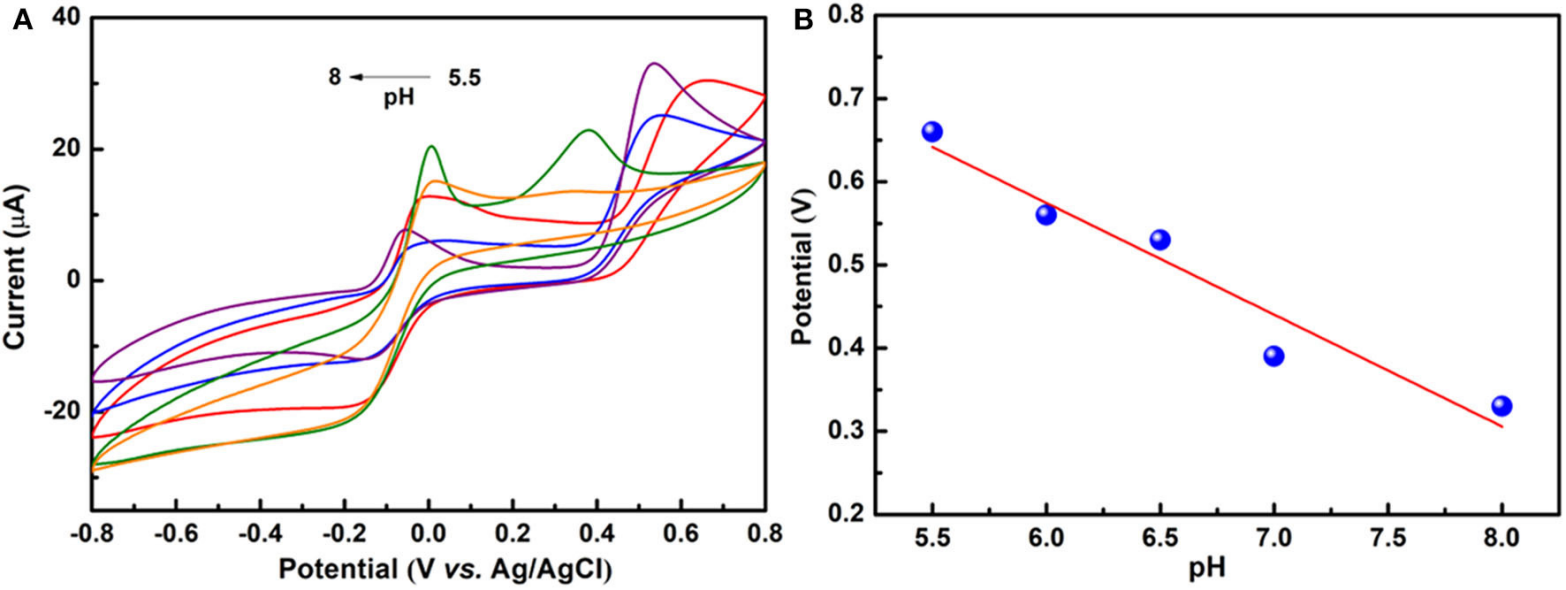
**(A)** CV curves of 50 μM paracetamol at the NiCu-CAT/GCE in 0.1 M PBS under 5.5, 6, 6.5, 7, 8 pH values. **(B)** The relationship between pH and peak potential.

Electrochemical properties were investigated with the conventional three-electrode system, as mentioned in the Methods and Materials section. The effect of pH on the peak potential of PA using cyclic-voltammetry in 0.1 M PBS at pH values of 5.5 to 8.0 is presented in Figure 3A. The effect of the pH value of the PBS on peak potential of 50 μM PA at NiCu-CAT/GCE is also investigated. It can be seen that with the pH increased, the oxidation peak shifted to a negative potential, indicating that this observation can be interpreted by observing the protons in the electrochemical reactions. The pH value 6.5 is chosen for further PA detection, with the highest sensitivity. In addition, Figure 3B shows that for a linear relationship between the peak potential (*E*_pa_) and pH value, the regression equation is shown by the following expression (Afkhami et al., 2004):

(1) $$\begin{array}{l} Epa\;(V)=Epa\left(pH=0\right)\;-\;\left(2.303mRT/nF\right)\;*pH \end{array}$$

where *E*_pa_(pH = 0) is the oxidation potential for paracetamol, R is the gas constant (8.314 J·K mol^−1^), F is Faraday's constant (96485 C mol^−1^), T is the Kelvin temperature (298.15 K), *n* is the number of electrons transferred, and *m* is the number of protons involved in the reaction. From Figure 3B, *E*_pa_ decreased with a slope of −47.5 mV/pH (*R* = 0.98802). Furthermore, from this equation, according to d*E*_pa_/dpH = −2.303 *m*RT/*n*F (Kang et al., 2010). Obviously, the redox reaction involves the same protons and number of electrons with a ratio of 1:1 (Fanjul-Bolado et al., 2009; Kalambate et al., 2015).

### Electrocatalytic Behavior of Paracetamol

**Figure 4:**
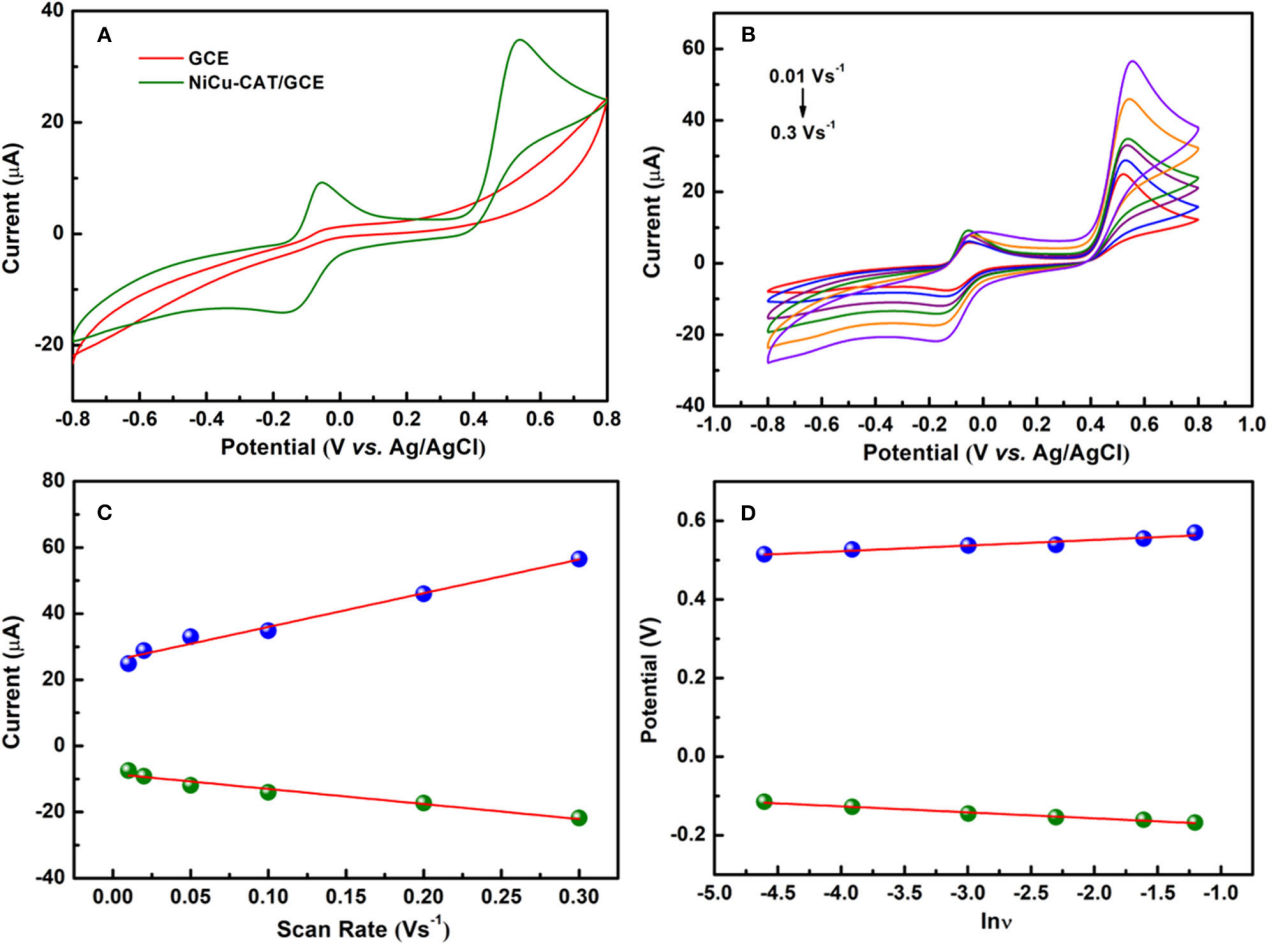
**(A)** CVs of GCE and NiCu-CAT/GCE in presence of 40 μM paracetamol. **(B)** CVs of 40 μM paracetamol on NiCu-CAT/GCE at scan rates of 10, 20, 50, 100, 200, and 300 mVs^−1^. **(C)** Displays the plot of the peak current vs. scan rates. **(D)** Displays the plot of the peak potential vs. lnν.

Cyclic voltammetry for NiCu-CAT/GCE is performed to investigate the electrochemical behavior of 40 μM paracetamol, electrodes are cycled between −0.8 and 0.8 V at a scan rate of 100 mV/s. Figure 4A shows CV responses of GCE and NiCu-CAT /GCE in PBS (pH = 6.5). No redox peaks are observed at GCE, which means that GCE is not electroactive in the studied potential region. In contrast, the redox peaks of NiCu-CAT/GCE appeared at −0.14 and 0.53 V, respectively, which can be attributed to the electrochemical redox process of the NiCu-CAT nanocrystals, and the current is larger than that of the bare glassy carbon electrode. The results show that NiCu-CAT has a good electrocatalytic effect on PA. Due to the special mesoporous structure of NiCu-CAT, the conductivity of the electrode is improved and the electrocatalytic effect of the electrode on PA is enhanced.

### Effect of the Potential Scan Rate

In order to investigate the mechanisms responsible for the oxidation of PA at NiCu-CAT/GCE, cyclic voltammograms of PA were recorded at various scan rates. It was observed that the cathodic peak current (*I*_pc_) and anodic peak current (*I*_pa_) increase linearly with the scan rate over the range of 10–300 mVs^−1^ for and PA. Figure 4B shows that the peak potential shifts forward with the increase of scanning rate. The linear relationship between the scan rate and peak current can be expressed by a linear regression equation (Raymundo-Pereira et al., 2016) as *I*_pa_[μA] = 101.88 ν[Vs^−1^] −0.25, (*R*^2^ = 0.980) and *I*_pc_[μA] = −45.45 ν[ Vs^−1^] −8.471 (R^2^ = 0.957) for the NiCu-CAT/GCE electrode, respectively (Figure 4C). The results show that PA undergoes an adsorption-controlled reaction (Goyal et al., 2010; Arvand and Gholizadeh, 2012; Kutluay and Aslanoglu, 2013).

To investigate the reaction kinetics, as shown in Figure 4D, the anodic and cathodic peak potentials have linear relationships with the natural logarithm of the scan rate (lnν). The linear regression equations are found to be:

(2) $$\begin{array}{l} \text{Epa }\left(\text{V}\right)=0.020ln\nu \;\left(\frac{V}{s}\right)\;+\;0.580,\;(R^{2}=0.9535) \end{array}$$

(3) $$\begin{array}{l} \text{Epc }\left(\text{V}\right)=-0.019ln\nu \;\left(\frac{V}{s}\right)\;-\;0.580,\;(R^{2}=0.9859) \end{array}$$

**Scheme 1:**
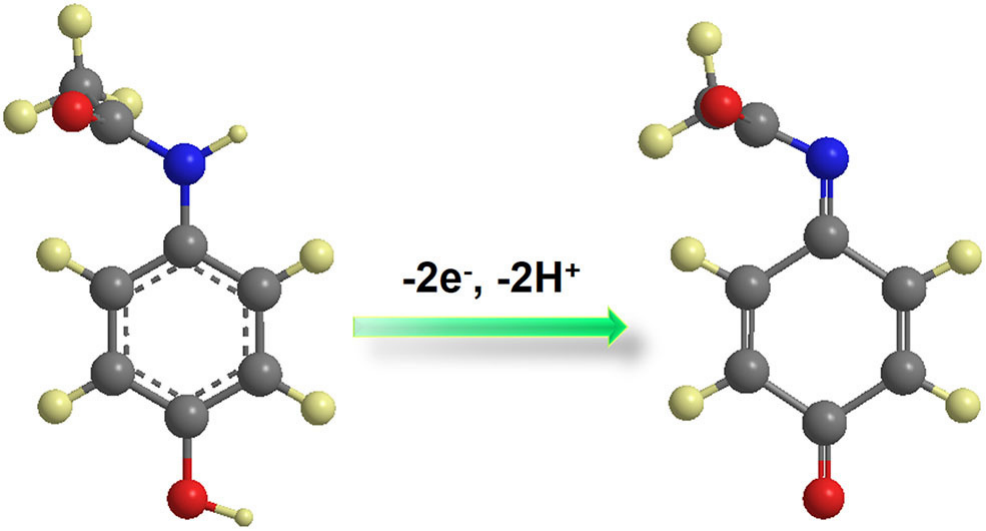
Redox mechanisms of NiCu-CAT/GCE.

According to Laviron's model (Laviron, 1974, 1979), the number of the electron–transfer (*n*) and charge-transfer coefficient (α) can be calculated to be 2 and 0.50, respectively. According to the above equations, results show that there is a two-proton and two-electron process for the PA electro-oxidation at NiCu-CAT/GCE. The possible redox mechanisms are as follows in Scheme 1.

### Analytical Performance Characteristics

**Figure 5:**
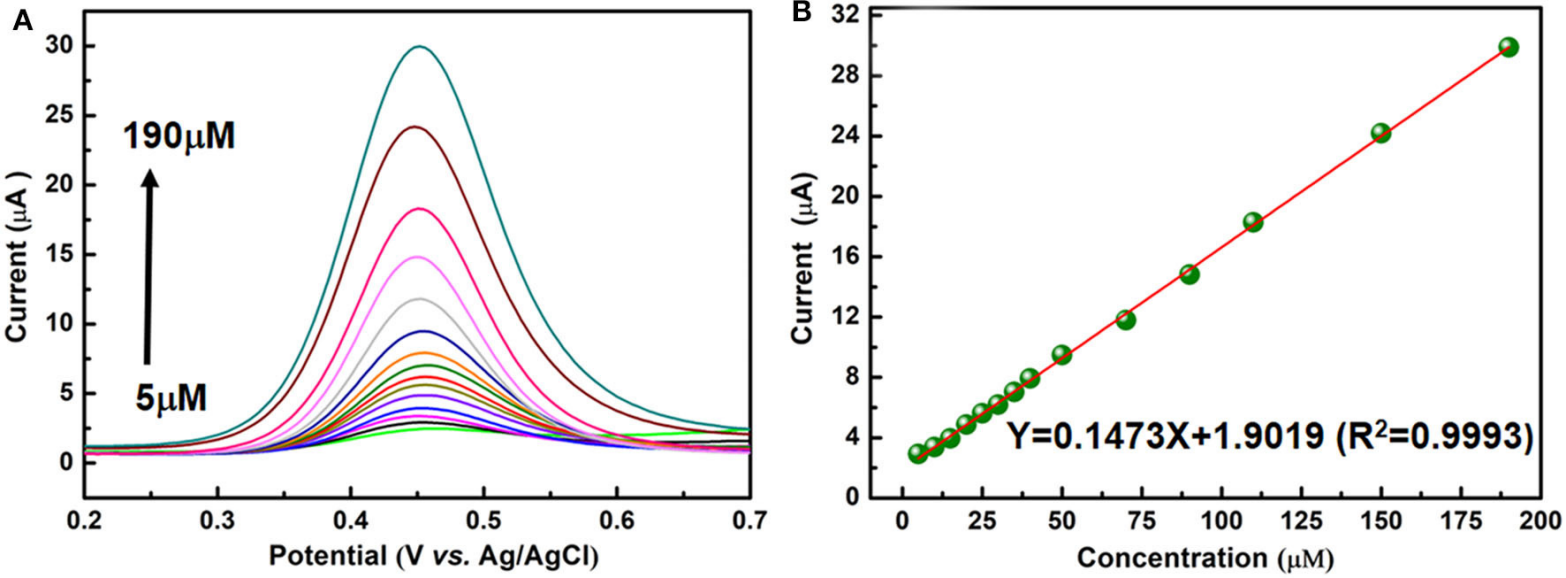
**(A)** DPV responses for different paracetamol concentrations (5, 10, 15, 20, 25, 30, 35, 40, 50, 70, 90, 110, 150, and 190 μM) on a NiCu-CAT/GCE. **(B)** The linear relationship between the peak current and paracetamol concentration.

DPV is utilized to measure the PA peak current on the present electrochemical sensor. This method has the advantages of high sensitivity and good resolution. Under optimized experimental conditions, the anodic peak current is directly proportional to the concentration of PA from 5 to 190 μM as shown in Figure 5A. From Figure 5B, it can be seen that the peak current of PA is in a linear dynamic range with its concentration. The regression equation (Goyal and Singh, 2006; Krampa et al., 2018; Xu et al., 2019) for the region is *I*_pa_(μA) = 0.1473*c*(μM) + 1.9019 (*R*^2^ = 0.9993), the limit of determination is near 5μM. The limit of determination of the NiCu-CAT/GCE is similar to some electrochemical sensors reported using electrochemical method for detecting paracetamol (Fu et al., 2015, 2018a,b). As shown in Supplementary Figure 7, the diameter of the paracetamol is 8.3 Å^*^ 3.7 Å, and the pore size of the Ni/Cu CAT is 13 Å. The paracetamol could easily go into the channel; consequently, we expected that the effects between the Ni/Cu CAT and paracetamol were based on host-guest interaction (Ko et al., 2017; Fang et al., 2018; Xie et al., 2020).

## Discussion

### Reproducibility, Stability and Interference

**Figure 6:**
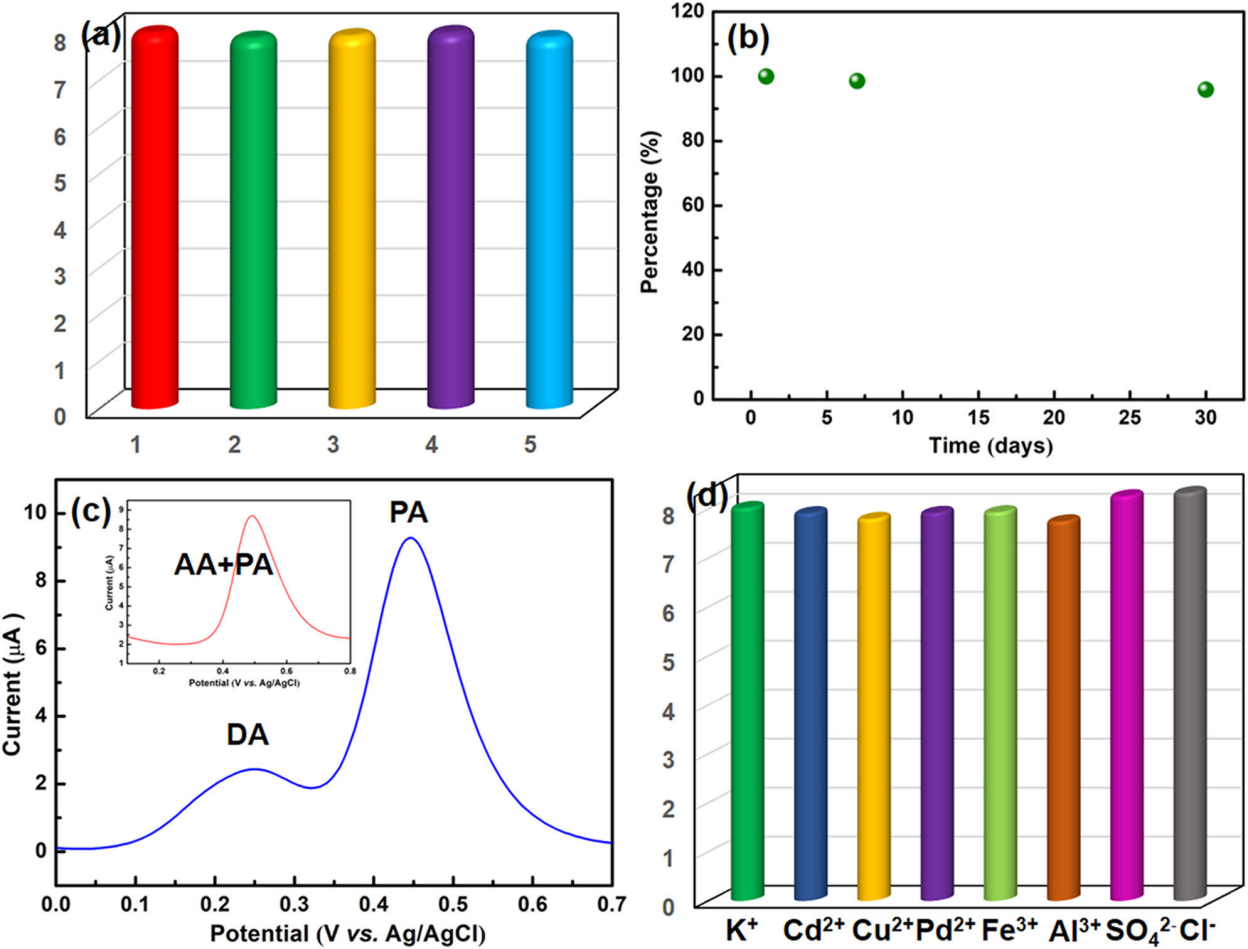
**(a)** Evaluation of repeatability. **(b)** Stability of the NiCu-CAT/GCE after pretreatment during 30 days. **(c)** DPV responses of the NiCu-CAT/GCE in PBS (0.1 M, pH 6.5) containing 40 μM PA and 200 μM DA; inset is the containing 40 μM PA and 200 μM AA, respectively. **(d)** Effects of the presence of inorganic ions on the voltammetric responses of 40 μM paracetamol using the NiCu-CAT/GCE.

The peak currents of five tests were recorded to study the reproducibility of the NiCu-CAT/GCE by DPV, and the same electrode was modified five times for PA detection at the same concentration of 40 μM (Figure 6a). Under the optimized conditions, the relative standard deviation (RSD) is 1.01%, indicating that NiCu-CAT/GCE can obtain satisfactory repeatability.

In order to study the stability of the modified GCE, the NiCu-CAT/GCE is used to measure 40 μM paracetamol in PBS (0.1 mol L^−1^, pH = 6.5) after being stored in the air for 7 days and 30 days, respectively (Figure 6b and Supplementary Figure 5). The fabricated sensors retain more than 95.8% of their original responses, indicating that NiCu-CAT/GCE has good stability. This stability is helpful to the application of chemically modified electrode in electroanalysis.

To investigate the sensor selectivity, the modified GCE is used to detect paracetamol in the presence of interferents. In addition, it was found that the peak current of 40 μM PA is not affected in the presence of 5-folds of dopamine (DA) and ascorbic acid (AA) (Figure 6c), and 100-fold excess concentrations of K^+^, Cd^2+^, Cu^2+^, Pb^2+^, Fe^3+^, Al^3+^, ${\text{SO}}_{4}^{2-}$, and Cl^−^ (Figure 6d). The results demonstrated that the potential interfering substances did not interfere with the 40 μM paracetamol signals, indicating that the present assay offers good sensitivity for determining paracetamol.

### Real Sample Analysis

In this paper, the NiCu-CAT electrochemical sensor was prepared to detect paracetamol in actual samples. The commercial tablet (Tylenol, produced in Shanghai, China) with a nominal value of 650 mg was used for the analysis of paracetamol. The tablets were pre-treated by grinding, dissolving with ethanol, filtering, and then diluting them with a phosphate buffer solution. The test results are shown in Supplementary Table 1. The recoveries of the tests were in the range from 97.23 to 103.8%. indicating that the modified electrode has a good detection performance for the actual samples containing PA, which is expected to be used for the detection of PA in real life.

## Conclusion

In summary, we have successfully constructed 2D conductive metal–organic frameworks as efficient electrocatalysts to achieve electrochemical detection of PA in aqueous solutions. The NiCu-CAT possesses a specifically big pore, numerous potential active sites, good electrical conductivity and water stability. The electrochemical properties of NiCu-CAT/GCE for PA were studied by cyclic voltammetry and the differential pulse method, under the optimal experimental conditions, the modified electrode has a wide linear range (5–190 μm) for the electrochemical detection of PA with good reproducibility and stability, and it also achieved the limit of determination near 5 μM. The research on the electrochemical detection of PA provides a platform for the application of MOF composites in electroanalysis, which is an excellent electrochemical method for pharmaceutical analysis.

## Data Availability Statement

All datasets generated for this study are included in the article/Supplementary Material.

## Author Contributions

JW executed the whole synthesis progress and paper writing. SL tested the samples of XRD and TEM. JL, SH, HS, YN, and CZ charged for data processing and result discussion. All authors contributed to the article and approved the submitted version.

## Conflict of Interest

The authors declare that the research was conducted in the absence of any commercial or financial relationships that could be construed as a potential conflict of interest.
