# Supplementary material for: Conductive Metal-Organic Frameworks for Amperometric Sensing of Paracetamol
Source: Front Chem. 2020 Dec 8;8:594093. doi: 10.3389/fchem.2020.594093 (PMC7793844; doi:10.3389/fchem.2020.594093)
Supplement: Supplementary file 1 [file Table_1.DOCX]

Supplementary Material

# Supplementary Figures and Tables

## Supplementary Figures


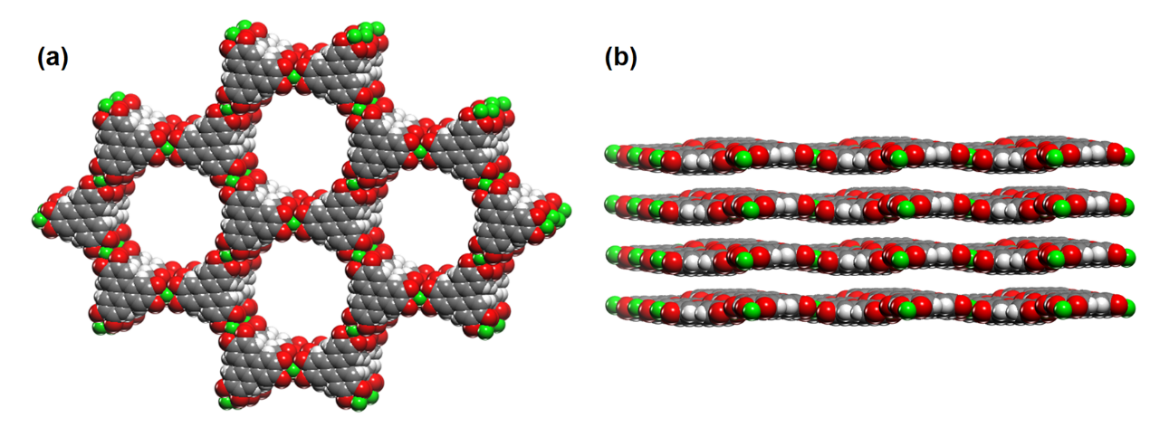


**Supplementary Figure 1.** (a) (b)Space filling drawings of the NiCu-CAT structure along the [001] and [110] direction.


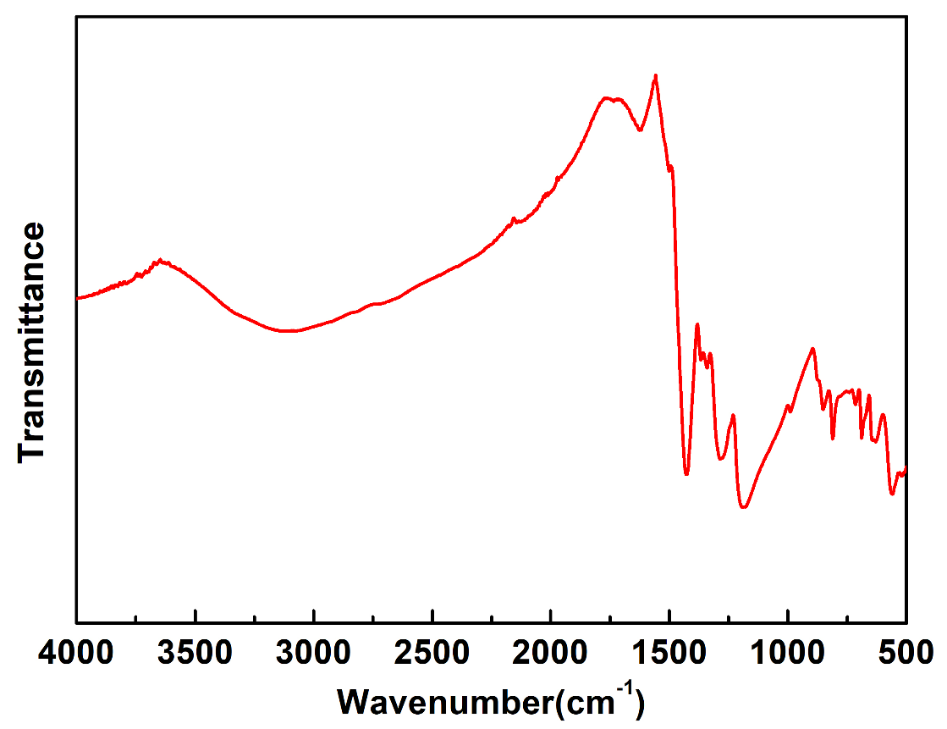


**Supplementary Figure 2.** FTIR spectra of NiCu-CAT.


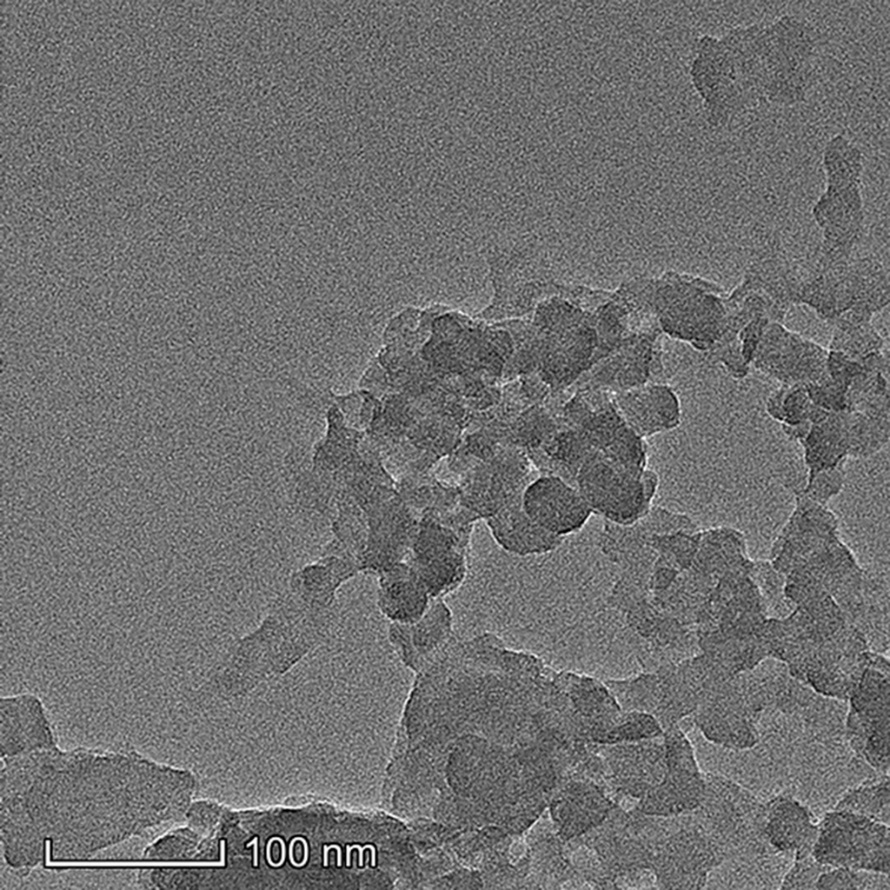


**Supplementary Figure 3.** TEM images.


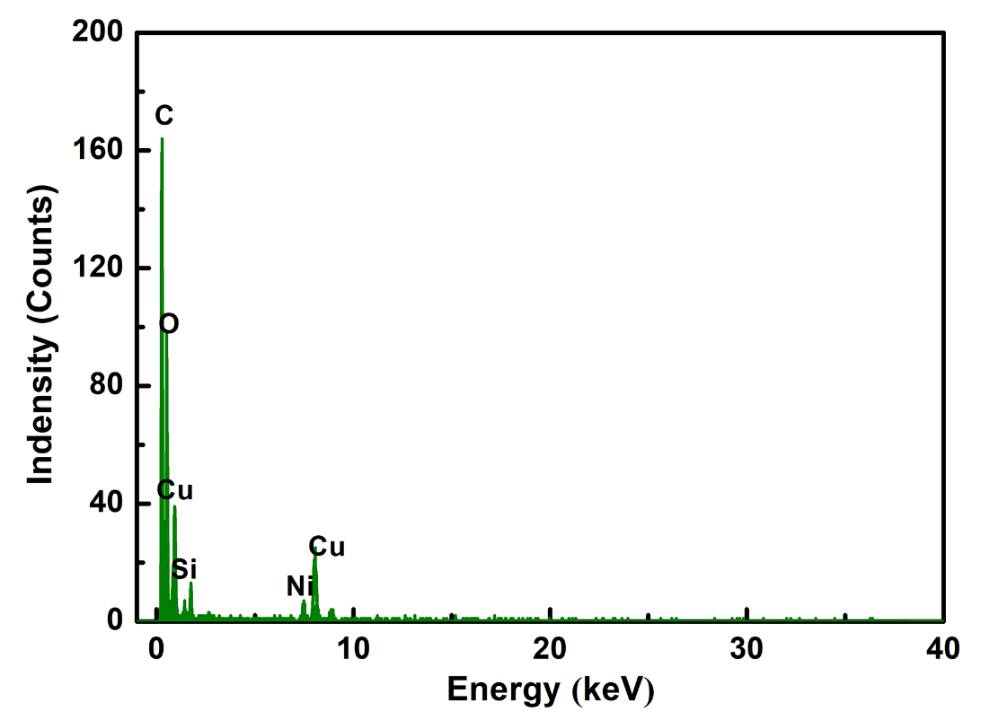


**Supplementary Figure 4.** EDX data of *NiCu-CAT*.
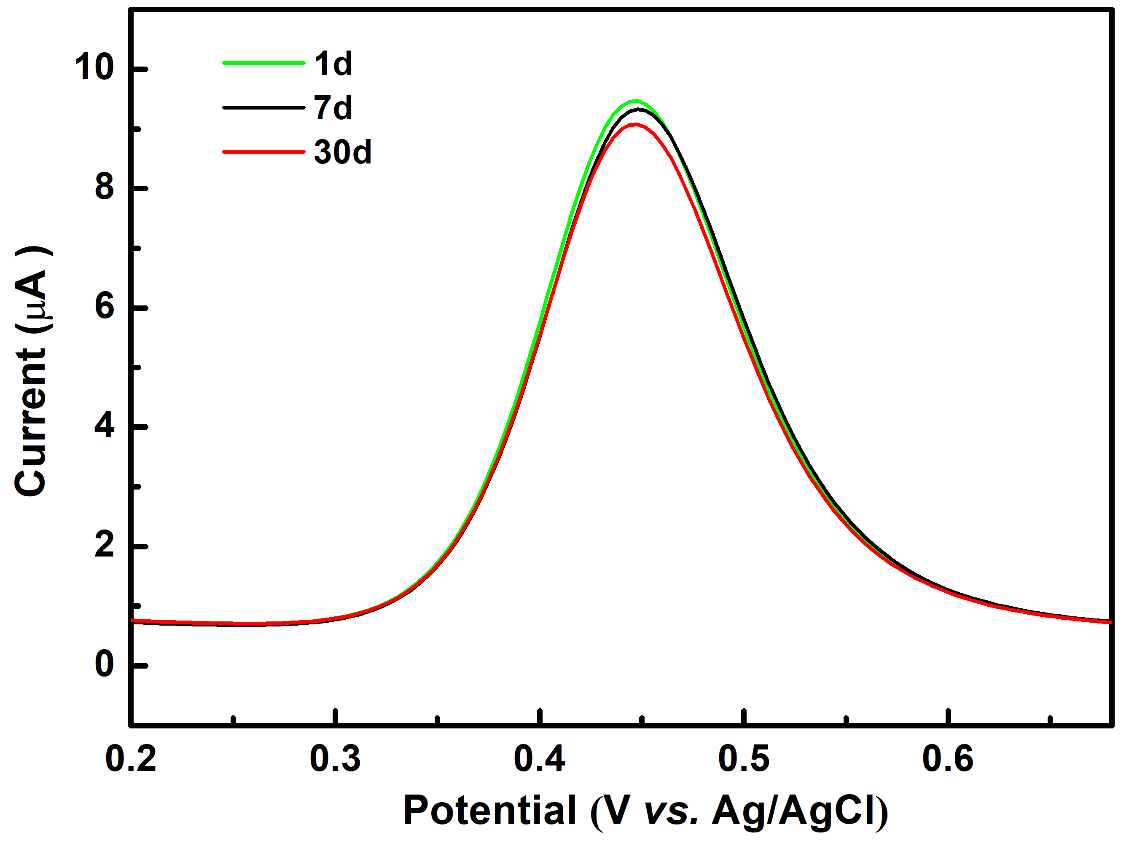


**Supplementary Figure 5.** DPV responses for different times.

**
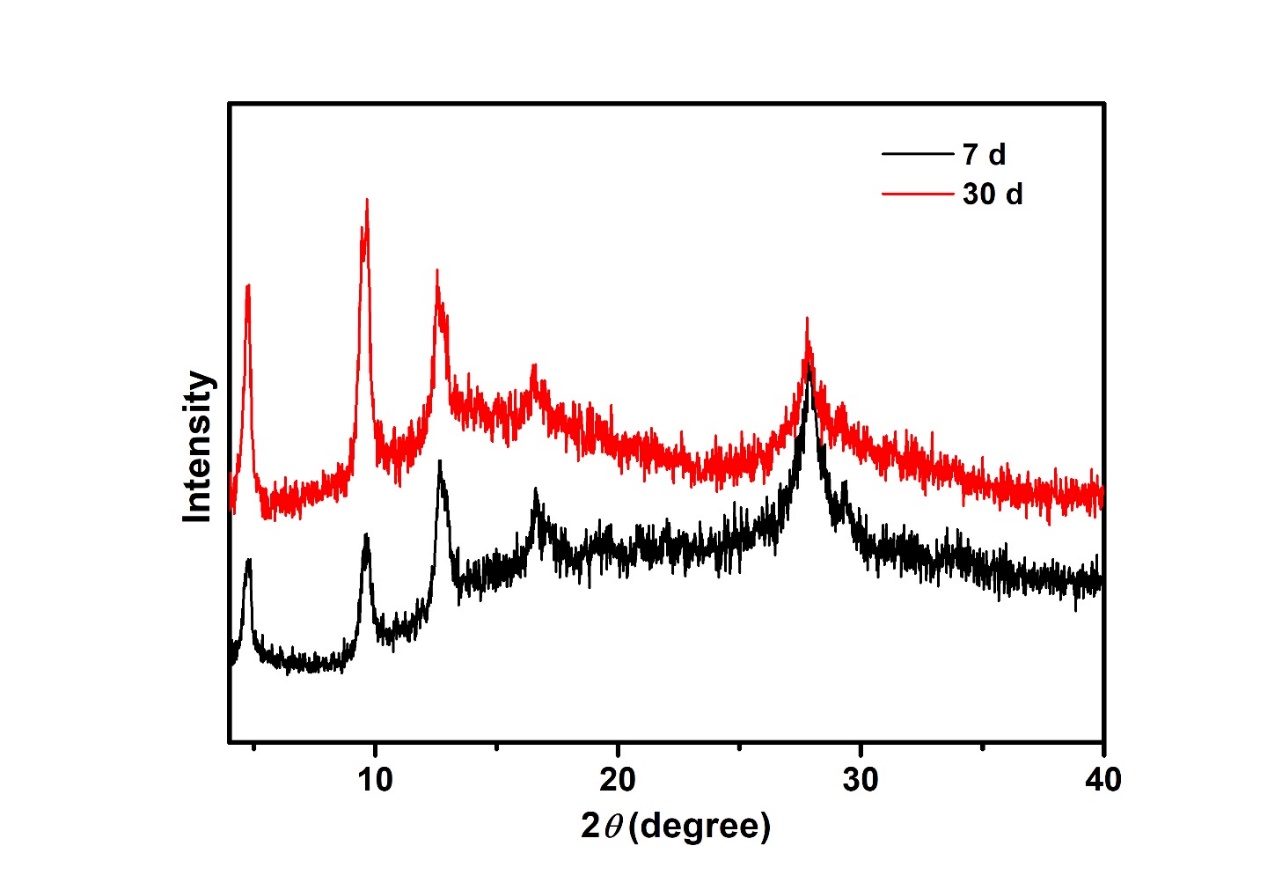
**

**Supplementary Figure 6.** XRD pattern after pretreatment during 30 days.


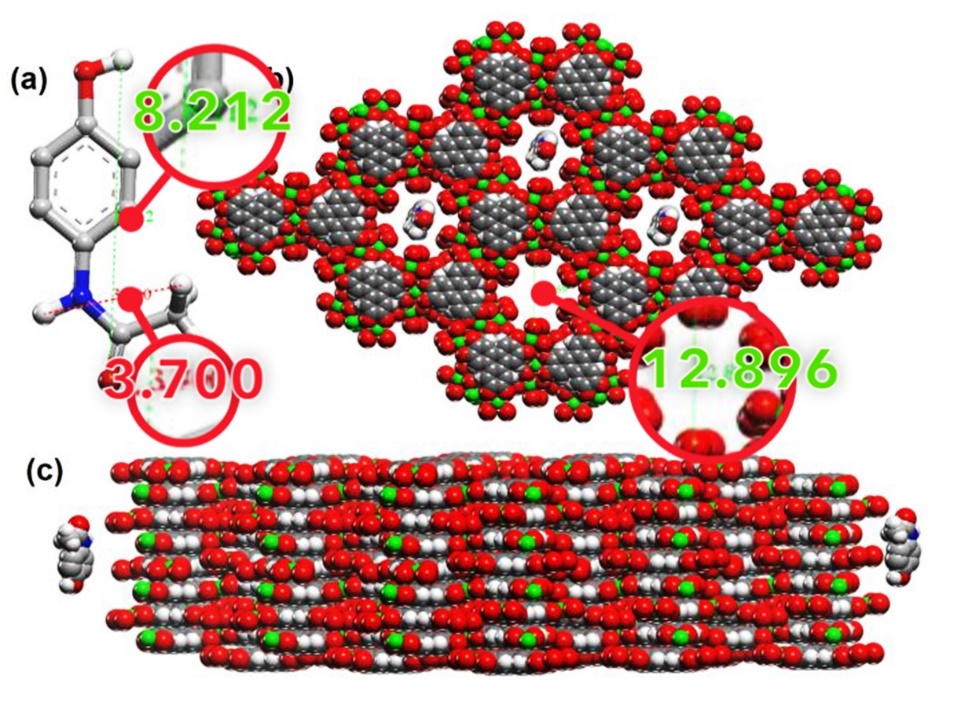


**Supplementary Figure 7.** (a) Paracetamol molecule, (b) (c) The relationship between Paracetamol molecule and CPK of NiCu-CAT structure along the [001] and [110] direction.

| No. | content (mg/   \| tablet) \| \| --- \| | Detection (mg/  tablet) | Recovery (%) |
| --- | --- | --- | --- | --- |
| Sample 1 | 650 | 640 | 98.46 |
| Sample 2 | 650 | 632 | 97.23 |
| Sample 3 | 650 | 675 | 103.8 |
| Sample 4 | 650 | 663 | 102.0 |

**Supplementary Table 1.** Determination of paracetamol in commercial tablets.
